# CD 56 staining in liver biopsies does not help in differentiating extrahepatic biliary atresia from other causes of neonatal cholestasis

**DOI:** 10.1186/1746-1596-3-10

**Published:** 2008-03-05

**Authors:** Fatemeh E Mahjoub, Reza Hadizadeh Khairkhah, Mehri Najafi Sani, Guiti Irvanloo, Maryam Monajemzadeh

**Affiliations:** 1 Assistant Professor in Pathology, Pathology Department, Markaze Tebbi Koodakan, Tehran University of Medical Sciences, Tehran, Iran; 2 Resident in Pathology, Pathology Department, Markaze Tebbi Koodakan, Tehran University of Medical Sciences, Tehran, Iran; 3 Associate Professor in Pediatric Gastroentrology, Gastroenterology Ward, Markaze Tebbi Koodakan, Tehran University of Medical Sciences, Tehran, Iran; 4 Assistant Professor in Pathology, Pathology Department, Imam Khomeini Hospital, Tehran University of Medical Sciences, Tehran, Iran; 5 Assistant Professor in Pathology, Pathology Department, Markaze Tebbi Koodakan, Tehran University of Medical Sciences, Tehran, Iran

## Abstract

**Introduction:**

Several conditions are considered in differential diagnosis of neonatal cholestasis. Of these the most important is extrahepatic biliary atresia (EHBA), while prompt diagnosis and surgical correction of obstruction can ameliorate clinical symptoms, provides long term survival for about one fourth of patients and serves as an important bridge to transplantation for many others. From histopathologic standpoint, features of EHBA overlaps with other diagnoses and so ancillary tests such as immunohistochemical staining for CD56 is suggested by some authors as a helpful tool in differential diagnosis.

Hereby we wanted to examine this staining in our center which is a referral children hospital and to prove its efficacy in our problematic cases.

**Materials and Methods:**

By retrospective review of pathology records during 2000 to 2006 in Markaze Tebbi Koodakan (children hospital related to Tehran University of Medical Sciences), we selected 17 cases of EHBA as patients and 12 cases with other diagnoses as controls, both with some degree of bile ductular proliferation in liver biopsies. EHBA cases were all proved by surgery. Four of control cases also underwent surgery but proved to have open ducts by intra-operative cholangiography. Long term follow up and other tests ruled out EHBA in other 8 cases. Hematoxylin-Eosin stains of paraffin blocks were studied again and freshly prepared sections were immunostained for CD56.

**Results:**

Bile ducts and proliferating bile ductules were strongly positive for CD56 in 6 of 17 cases of EHBA. In 7 out of 17, positivity were seen in more than two thirds of portal tracts. In controls, one case showed strong positivity and 6 out of twelve showed positivity in more than two thirds of portal tracts. The intensity and distribution of CD56 staining did not differ significantly between two groups.

**Discussion:**

Despite findings of previous studies, we have shown that CD56 staining can not help as an ancillary test in differential diagnosis of neonatal cholestasis and perhaps other markers should be tested in this regard.

## Introduction

Extrahepatic biliary atresia is the most common cause of pathologic infant jaundice and one of the most common reasons for liver transplantation in children [1]. It is a condition in which there is total or segmental obliteration of the extrahepatic duct system [2]. Several reports suggest that it results from prenatal injury and post-inflammatory fibrous obliteration of extra hepatic biliary tree [2]. Several infectious agents such as reovirus 3, rotavirus C, rubella and cytomegalovirus are suggested as causative agents [3]. Classically, a 1 to 2 month old child presents with clay colored stools, jaundice, lack of bile excretion on cholecintigraphy by hepatobiliary iminodiacetic acid (HIDA) and a liver biopsy specimen that shows changes of extrahepatic biliary tract disease with fibrous expansion of portal tracts and marked bile ductular proliferation [1]. In many cases, the clinical and radiographic findings are not diagnostic and histologic findings are critical in patient management decisions. The histologic findings of EHBA vary depending on when the biopsy is obtained in the course of the disease. Liver biopsies obtained early in the course of the disease typically in children younger than 80 days demonstrate downstream obstructive type changes with ductular proliferation, variable portal edema, and lobular cholestasis. Other nonspecific changes such as focal multinucleated giant hepatocytes are also seen. Biopsies taken later in the course of the disease typically show less ductular proliferation and may show ductopenia [4,5]. The most useful distinguishing feature is the portal fibrosis and bile duct proliferation in biliary atresia, although other causes of obstruction and also some cases of nonobstructive cholestasis may show a similar pattern such as alpha 1 anti-trypsin deficiency, cystic fibrosis and progressive familial intrahepatic cholestasis type III [2,6]. Because of this it is necessary to prove EHBA by ancillary methods such as immunostaining by CD56 which is reported as helpful aid in differentiating various causes of neonatal cholestasis [1]. CD56 or N-CAM is an isoform of neural cell adhesion molecule that is commonly used as a marker of natural killer cells but is expressed in a variety of normal tissues and also tumors such as thyroid carcinoma and renal cell carcinoma [7]. It is not normally expressed in biliary epithelium, but is claimed to be strongly expressed in the setting of obstructive biliary tract disease [8,9].

The aim of our study was to examine this claim by a case control study in patients under three months of age who referred with cholestasis.

## Materials and Methods

Pathologic records of patients under 3 months of age were retrospectively reviewed to identify all cases diagnosed as EHBA from 2000–2006 in Markaze Tebbi Koodakan (children hospital related to Tehran University of Medical Sciences) as well as cases in which biliary atresia was in the clinical and histological differential diagnosis. We found 191 cases who referred with cholestasis and underwent liver biopsy, in these we selected 58 cases with final pathologic report of "compatible with large bile duct obstruction" or cases with some degree of bile ductular proliferation. 29 cases were excluded due to variety of causes such as incomplete follow up and inadequate specimen. Also patients records were reviewed for clinical and paraclinical data as presence of acholoic stool, hepatosplenomegaly, results of HIDA scan and liver and bile duct sonography.

All tissues were fixed in 10% neutral buffered formalin. Five micron sections from the paraffin embedded tissue (fresh sections were cut for the immunohistochemical staining as recommended by Torbenson et al [1], because immunoreactivity of CD56 diminishes in unstained archival sections kept at room temperature) were stained with CD56 (clone 1B6, ready to use, Novocastra, Newcastle, UK) using the Novocastra detection system (RE 7150-K) following heat antigen retrieval by microwave in citrate (pH: 6). Incubation time of antibody was 30 minutes. All the slides were seen by one pathologist (F. Mahjoub). Staining in the interlobular bile ducts and ductules was evaluated for distribution on a scale of 0 to 3 (as recommended by Torbenson et al) (Table 1). Staining intensity was also scored on a scale of 0 to 3 (Table 1).

**Table 1:** Staining in the interlobular bile ducts and ductules was evaluated for distribution and intensity on a scale of 0 to 3 (as recommended by Torbenson et al), depicted in this table.

	**0**	**1**	**2**	**3**
Distribution	No staining	Any staining in less than one third of portal areas	Staining in one third to less than two thirds of portal areas	Staining in more than two thirds of portal areas
Intensity	No staining	Any weak staining	Moderate staining	Strong staining

Kupffer cells were served as positive control.

The data were collected and processed by Chi-square and Fischer's exact tests in SPSS software. A P-value of 0.05 or less was considered significant.

## Results

**Figure 1:**
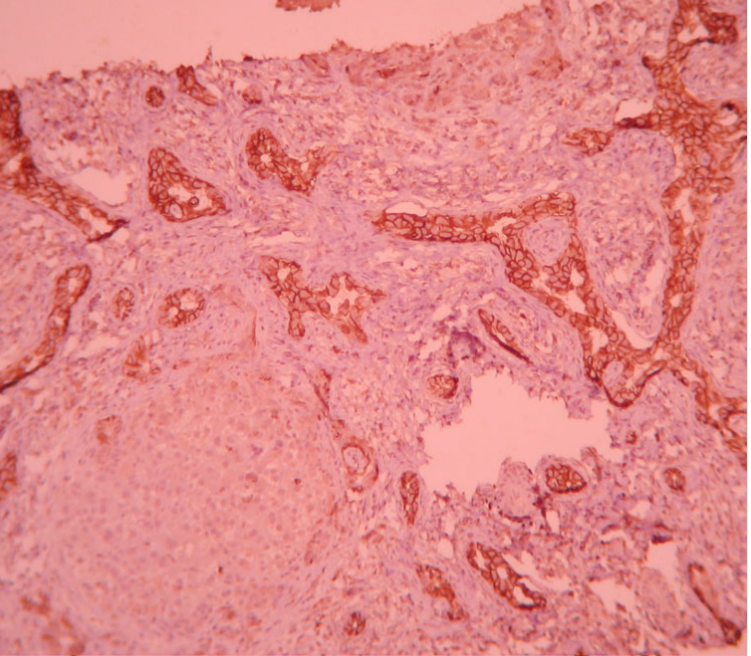
CD56 immuno-staining: strong positive staining (3) in more than two third of portal areas (3). (×100).

**Figure 2:**
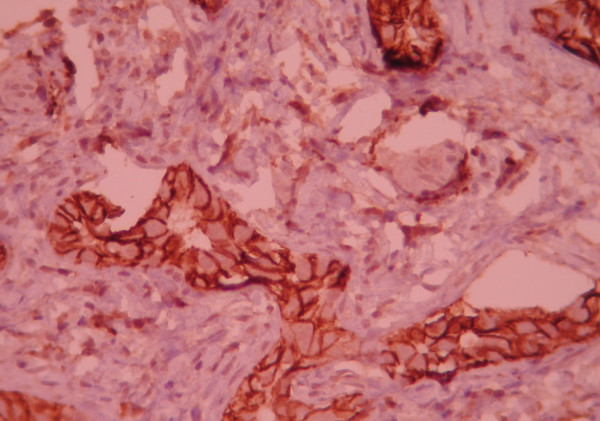
closer view of picture one (×400).

**Figure 3:**
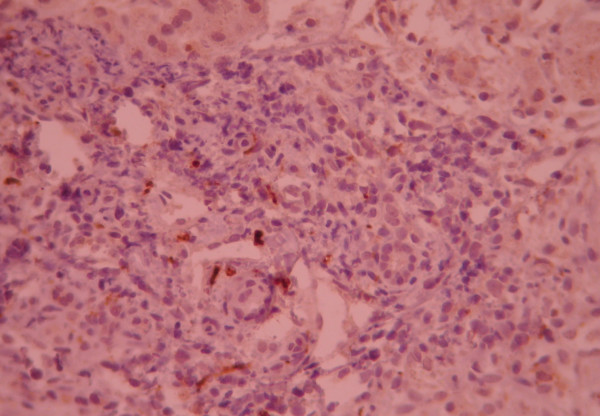
Negative CD 56 immuno-staining, only lymphoid cells are stained and bile ducts and ductuli remain unstained.

A total of 29 cases were included in the study: seventeen cases of confirmed biliary atresia and 12 cases of other conditions consisting of: three cases with final diagnosis of progressive intrahepatic cholestasis type III (PFIC, III), three cases with final diagnosis of self-limited cholestatic hepatitis, two cases of cystic fibrosis (CF), two cases of inborn errors of metabolic diseases and two cases of neonatal giant cell hepatitis. Controls were intentionally selected to have some degree of bile ductular proliferation, and in all cases extrahepatic biliary atresia was in the histologic differential diagnosis. All cases had at least five portal tracts. Strong positivity for CD56 was detected in 6 of 17 (35.3%) cases of EHBA in both bile ducts and proliferating ductules (score 3) (Table 2). In 7 of 17 cases (41.2%) more than two thirds of portal tracts showed positive staining ducts and ductules (score 3) (Fig: 1 &2). Two cases were negative. Two out of 12 control cases were completely negative, including one case of neonatal (giant cell) hepatitis and one case of PFIC type III (Fig 3). One case of cystic fibrosis showed strong positivity (score 3). Other control cases showed positivity with intensity ranging from 1 to 2 and distribution score of 1 to 3. Staining intensity and distribution does not show significant difference between controls and cases.

**Table 2:** Diagnosis of patient in relation to staining intensity and distribution for CD56.

**No**	**Diagnosis**	**Intensity**	**Distribution**
1	Biliary atresia	1	1
2	Biliary atresia	2	2
3	Biliary atresia	3	3
4	Biliary atresia	2	2
5	Biliary atresia	2	2
6	Biliary atresia	3	3
7	Biliary atresia	2	2
8	Biliary atresia	0	0
9	Biliary atresia	2	3
10	Biliary atresia	1	2
11	Biliary atresia	3	3
12	Biliary atresia	3	3
13	Biliary atresia	3	3
14	Biliary atresia	0	0
15	Biliary atresia	3	1
16	Biliary atresia	2	3
17	Biliary atresia	1	1
18	PFIC, TYPE III	1	2
19	PFIC, TYPE III	1	1
20	PFIC, TYPE III	1	2
21	Cholestatic hepatitis of unknown etiology	1	2
22	Cholestatic hepatitis of unknown etiology	0	0
23	Cholestatic hepatitis of unknown etiology	2	3
24	Cystic fibrosis	3	3
25	Cystic fibrosis	1	3
26	Inborn error of metabolism	2	3
27	Inborn error of metabolism	2	3
28	Neonatal (Giant cell) hepatitis	2	3
29	Neonatal (Giant cell) hepatitis	0	0

## Discussion

Extrahepatic biliary atresia is the most common cause of pathologic infant jaundice and one of the most common reasons for liver transplantation in children. The diagnosis of EHBA can be challenging as the histologic features can overlap other pediatric cholestatic liver diseases [1]. In our center we encountered several cases with cholestasis and histologic features resembling EHBA and confirmation by sonography and HIDA scan which were proved to have patent biliary ducts during surgery. So we decided to review our cases and also test CD56 staining that is reported by Torbenson et al to be useful for diagnosis of EHBA in early ductular proliferative phase. The histopathology of EHBA is essentially that of obstructive extrahepatic biliary tract disease. Classically, biopsy specimens show an extensive ductular proliferation, with elongated and agulated ductules that occasionally contain bile plugs. Ductopenia can be seen in biopsies taken later in the course of the disease, usually in children older than four months [4,5]. So we decided to study this immunostain only in children less than 3 months of age. The main differential diagnosis of EHBA are progressive familial intrahepatic cholestasis type III, neonatal hepatitis (giant cell) and cystic fibrosis. Although those with cystic fibrosis may benefit surgery and washing of inspissated bile plugs, in the other two conditions surgical operation may be harmful, hence definite diagnosis especially by histological means prior to surgery is critical in these patients. CD56 is an isoform of N-CAM that serves as an adhesion molecule for neural cells as well as for natural killer cells. In addition, it appears that CD56 is upregulated in the biliary tree in situations of extrahepatic biliary tract disease. Interestingly, other adhesion markers such as ICAM-1 and VCAM are also upregulated in EHBA, although they were neither exclusive to nor characteristic of EHBA in Davenport study [10]. CD56 also strongly stains bile ducts in the setting of alcoholic liver disease [11], focal nodular hyperplasia [12] and congenital hepatic fibrosis [13]. Also it commonly stains bile ducts and proliferating ductules in the setting of TPN (total parenteral nutrition) therapy [8].

In our study a total of 29 cases were included, all under 3 months of age (although in Torbenson's study older children were also included), 17 with proved EHBA by surgical means (14 in Torbenson's study, all proved as ours), besides we chose cases having needle biopsies rather than wedge biopsies (which was also included in Torbenson's study). 12 controls were selected (8 in Torbenson's study), 4 proved to have patent ducts by surgery, and in the remainder EHBA was ruled out by other means (such as follow up of patients which revealed a self limited course, positive sweat test for two times and metabolic tests). Torbenson reported that bile ducts were positive for CD56 in 13 of 14 cases of EHBA, both bile ducts and proliferating ductules showed positivity and the staining intensity was generally strong and in more than two thirds of portal tracts [1]. However in our study strong positivity was seen in only 6 of 17 patients with proven EHBA and in one of 12 control cases which was not significant statistically (p value: 0.18). Distribution of positive staining in EHBA was in more than two thirds of portal areas in 7 cases from 17 and in 6 from 12 control cases which was not significant statistically (p value: 0.6). Differences between two studies are depicted in Table 3.

**Table 3:** Comparison of Torbenson results and our findings. P value of intensity and distribution between two studies were 0.18 and 0.6 which was not significant statistically.

**Torbenson's Article**			**Our Data**		
CD56 Staining (Number of Cases)			CD56 Staining (Number of Cases)		

Cases		Controls	Cases		Controls

Neg	1 (small biopsy)	4	Neg	2	2

Pos	13, mostly strong and in more than two thirds of portal tracts	3: patchy and weak	Pos	15: only 6 strong and only 7 cases with positivity in more than two thirds of portal tracts.	12: one strong and 6 cases with positivity in more than two thirds of portal tracts.

We suppose that maybe selection of patients in two studies (Torbenson's group included older children also), technical differences and use of both wedge and needle biopsies by Torbenson has resulted in different findings. Although difference between two groups may become more prominent on wedge biopsies, we chose only needle biopsies while we wanted to assess the efficacy of this method prior to surgical exploration which may be harmful in non EHBA cases.

In summary we conclude that despite Torbenson's results about utility of CD56 staining in differentiating EHBA from other causes, better means for this purpose should be searched for.
